# Protein Intake and Physical Activity in Newly Diagnosed Patients with Acute Coronary Syndrome: A 5-Year Longitudinal Study

**DOI:** 10.3390/nu13020634

**Published:** 2021-02-16

**Authors:** Andrea Greco, Agostino Brugnera, Roberta Adorni, Marco D’Addario, Francesco Fattirolli, Cristina Franzelli, Cristina Giannattasio, Alessandro Maloberti, Francesco Zanatta, Patrizia Steca

**Affiliations:** 1 Department of Human and Social Sciences, University of Bergamo, 24129 Bergamo, Italy; agostino.brugnera@unibg.it; 2 Department of Psychology, University of Milano-Bicocca, 20126 Milan, Italy; roberta.adorni1@unimib.it (R.A.); marco.daddario@unimib.it (M.D.); francesco.zanatta@unimib.it (F.Z.); patrizia.steca@unimib.it (P.S.); 3 Department of Medical and Surgical Critical Care, Cardiac Rehabilitation Unit, University of Florence, 50139 Florence, Italy; francesco.fattirolli@unifi.it; 4 Azienda Ospedaliero-Universitaria Careggi, 50134 Florence, Italy; 5 Cardiac Rehabilitation Centre-CTO Hospital, 20126 Milan, Italy; cristina.franzelli@asst-pini-cto.it; 6 School of Medicine, Surgery University of Milano-Bicocca, 20126 Milan, Italy; cristina.giannattasio@unimib.it (C.G.); alessandro.maloberti@ospedaleniguarda.it (A.M.); 7 Cardiology IV, “A. De Gasperis” Department, Ospedale Niguarda Ca’ Granda, 20162 Milan, Italy

**Keywords:** acute coronary syndrome, healthy behaviors, diet, legumes, fish, red/processed meat, physical activity, anxiety, depression, season

## Abstract

Cardiovascular disease is one of the most common causes of hospitalization and is associated with high morbidity and mortality rates. Among the most important modifiable and well-known risk factors are an unhealthy diet and sedentary lifestyle. Nevertheless, adherence to healthy lifestyle regimes is poor. The present study examined longitudinal trajectories (pre-event, 6-, 12-, 24-, 36-, and 60-month follow-ups) of protein intake (fish, legumes, red/processed meat) and physical activity in 275 newly-diagnosed patients with acute coronary syndrome. Hierarchical Generalized Linear Models were performed, controlling for demographic and clinical variables, the season in which each assessment was made, and the presence of anxiety and depressive symptoms. Significant changes in protein intake and physical activity were found from pre-event to the six-month follow-up, suggesting the adoption of healthier behaviors. However, soon after the six-month follow-up, patients experienced significant declines in their healthy behaviors. Both physical activity and red/processed meat intake were modulated by the season in which the assessments took place and by anxiety symptoms over time. The negative long-term trajectory of healthy behaviors suggests that tailored interventions are needed that sustain patients’ capabilities to self-regulate their behaviors over time and consider patient preference in function of season.

## 1. Introduction

Cardiovascular disease (CVD) is one of the most common causes of hospitalization in Western countries and is associated with high morbidity and mortality rates. CVD’s burden is not only a health issue, but also a growing economic and societal challenge [1,2]. INTERHEART [3], a case-control study conducted in 52 countries, has identified nine risk factors and health behaviors (namely, hypertension, dyslipidemia, diabetes, obesity, smoking, alcohol, unhealthy diet, sedentary lifestyle, and psychosocial factors) that account for more than 90% of the population attributable risk of CVD. Based on scientific evidence that the virtuous management of these risk factors may reduce the incidence of CVD at the population level, eight of them (all except psychosocial factors) are the World Health Organization targets for reduction by 2025 [4]. Nevertheless, according to a recent report from the European Society of Cardiology [2], based on current trends, only the reduction in smoking from 28% to 21% over the last 20 years appears to be able to meet the WHO target. This goal may be achieved for smoking, but other behaviors like unhealthy diet and sedentary lifestyle still need strong attention.

Regarding physical activity, international guidelines recommend that adults engage in at least 150 minutes per week of accumulated moderate-intensity or 75 minutes per week of vigorous-intensity aerobic physical activity to reduce CVD risk [1,2]. Encouraging leisure-time exercise has consistently been shown to promote cardiovascular health [5]. International guidelines recommend a diet emphasizing the intake of fruits and vegetables, whole grains, fish, and legumes, and minimizing the intake of processed meats and fats to decrease CVD risk factors [1]. The widespread popularity of high-protein diets has drawn controversy as well as scientific interest [6]. Many meta-analyses have shown a potential CVD benefit for mainly secondary prevention with increased fish intake [7] and decreased red/processed meat intake [8]. Data about the intake of legumes are less consistent but go in the same direction [9,10].

Despite the large amount of scientific evidence and recommendations described in the international guidelines, both in terms of primary and secondary prevention, there is poor compliance with regimens of a healthy diet and physical activity [5]. Studies focused on the longitudinal trajectories of healthy lifestyle highlighted that patients with established CVD after the initial adoption of healthier lifestyles tend to drop out within six months of hospital discharge [11,12,13].

The role of psychological factors is largely underestimated. Indeed, these risk factors are not currently recorded in the ESC Atlas [2], nor are they among the WHO’s targets for management for 2025 [4], although they are well established as contributors to CVD risk [14,15,16]. In a previous study by the present research group, an association between lifestyle profiles and psychological factors of depression and anxiety was found [17], consistent with other empirical evidence showing a deleterious effect of depression and anxiety on changing unhealthy lifestyles [18]. In another study [19], we found that higher levels of depression six months after an acute coronary event were associated with subsequent unhealthy lifestyle six months later. However, higher levels of depression at baseline were not associated with subsequent unhealthy lifestyle six or twelve months later. These findings underscore the importance of investigating the long-term role of psychological factors in predicting healthy behavior trajectories that to date remain almost unknown.

Compliance with regimens of a healthy lifestyle may also be influenced, at least partially, by the seasons [20]. Indeed, bad weather can represent a barrier to carrying out physical activity, especially outdoors. Again, eating habits change considerably according to the season [21,22]. All these changes, by modifying physiological responses and metabolism, could affect cardiovascular function and disease [23]. These changes may be particularly pronounced in countries subject to four distinct seasons and with very different winter-to-summer weather conditions, like countries in the Mediterranean area. Nevertheless, seasonal variations have seldom been considered a factor that modulates compliance with healthy regimens in patients with established CVD [24,25].

The present study explores the longitudinal trajectories and underlying predictors associated with protein consumption and physical activity in a cohort of 275 consecutive patients with acute coronary syndrome (ACS) at their first coronary event. The aim was to assess whether these factors could play a significant role in predicting adherence to physicians’ prescriptions to achieve and maintain a sufficient level of physical activity and a balanced protein intake. In addition to the most well-known demographic and clinical predictors, for the first time, this study has investigated the role played by both psychological factors, namely anxiety and depressive symptoms, and environmental factors, i.e., seasonal variation. Patients were assessed at six time points, i.e., at baseline, at six months, and at one, two, three, and five years after the first cardiovascular event. An advanced statistical technique, namely hierarchical linear models (HLMs) with a piecewise regression approach, was used to model the individual change rate from pre-event to five-year post-cardiovascular event. This technique allowed us to test the hypotheses that (1) patients experienced increases in healthy protein consumption and physical activity from pre- to six-months post-cardiovascular event; (2) patients decreased their healthy behaviors from six-months to five-years post-cardiovascular event. We trust that investigating the predictors of lifestyle changes in patients affected by CVD can be a useful tool for developing tailored cardiovascular rehabilitation programs to increase and stabilize healthy behaviors. This may have a great impact on the effectiveness of healthcare practices.

## 2. Materials and Methods 

### 2.1. Study Design and Participants

A total of 275 consecutive patients affected by ACS at their first coronary event were recruited from February 2011 to October 2013, in three large public hospitals in Italy. Eligible patients were between 30 and 80 years of age, had sufficient Italian language skills, and had neither cognitive deficits nor comorbidity with other major pathologies such as cancer. Physicians recruited patients who met the eligibility criteria during their cardiovascular rehabilitation (CR) at the hospital, which took place between two and eight weeks after their first acute coronary event. Information on lifestyle before the onset of ACS was retrospectively collected during the first assessment (baseline). After the first data collection, patients were re-evaluated at five subsequent follow-ups (after six months, one, two, three, and five years). At each time-point, sociodemographic, clinical, psychological, and behavioral data were collected. The date of each of the six assessments was used to define the variable “season” at each time-point.

This study is part of a larger longitudinal study aimed at profiling patients with ACS and hypertension in terms of a series of behavioral, clinical, and psychological variables [13,17,19,26]. For the first time, this study has focused on a five-year period after the first cardiovascular event and has considered the role of both psychological (anxiety and depressive symptoms) and environmental (seasonal variations) factors as predictors of the longitudinal trajectories of healthy behaviors in terms of diet and physical activity.

The Bio-Ethics Committee of all the institutions involved in the research project approved the study. Each participant provided written informed consent before their enrollment.

### 2.2. Measures

#### 2.2.1. Protein Consumption

Protein consumption was measured using three items (pertinent to fish, legumes, and red/processed meat intake) from the Italian version of the Mediterranean Diet Scale (MDS) [26,27]. The MDS is a nine-item self-report questionnaire that measures the weekly consumption of nine foods using a six-point Likert scale (from 1 = Never to 6 = More than three times per day). Each response was coded as a dichotomous variable following Trichopoulou and collaborators [27]; 1 indicates healthy (two or more servings of fish per week; two or more servings of legumes per week; two or fewer servings of red/processed meat per week) and 0 indicates unhealthy consumption.

#### 2.2.2. Physical Activity

Physical activity was measured using the Italian version of the Rapid Assessment of Physical Activity Questionnaire (RAPA-Q) [28], a seven-item measure of the frequency and intensity of the participants’ physical activity. The questionnaire uses a yes/no scale. The total score ranges from 1 (i.e., sedentary) to 7 (i.e., regular and vigorous activity), with higher scores indicating a healthier amount of physical activity. Scores of 6 or 7 (i.e., at least 30 min of moderate to vigorous aerobic exercise five times a week) indicate the target amount of physical activity for cardiovascular prevention.

#### 2.2.3. Anxious and Depressive Symptoms

Anxious and depressive symptoms were measured using the Italian version of the Hospital Anxiety and Depression Scale (HADS) [29,30], a 14-item self-report questionnaire developed to screen for generalized symptoms of psychological distress in medical patients. Participants reported their feelings and mood on a four-point Likert scale (for example, “I’ve lost interest in my appearance”, and the possible answers are 3 = “definitely”, 2 = “I don’t take as much care as I should”, 1 = “I may not take quite as much care”, and 0 = “I take just as much care as ever”). Two sum scores were calculated for anxiety and depressive symptoms; the total score ranges from 0 to 21, in which higher scores indicate a greater presence of symptoms. The scale showed adequate internal consistency (Cronbach’s alphas: anxiety = 0.81, depression = 0.73).

### 2.3. Data Analysis

Levels of physical activity from pre-event to the five-year follow-up were evaluated using two-level Hierarchical Linear Models (HLMs), whilst changes in fish, legume, and red/processed meat consumption (three dichotomous variables) were analyzed using hierarchical generalized linear models (H(G)LMs). H(G)LMs are the best statistical methods to examine longitudinal changes in nested data [31]. Their main advantage is their flexibility in handling missing data [32], a common occurrence in longitudinal studies.

We tested the hypotheses on lifestyle behaviors using piecewise regression models in H(G)LM, in which two level-1 “time” parameters were included to model the slope discontinuity from pre- to six-months post-cardiovascular event (Time.D1), and from six-months to five-year post-cardiovascular event (Time.D2) [33].

H(G)LM analyses were adjusted for several confounding demographic and clinical variables, namely age, sex, working status (not working vs. working), educational level (less than high school vs. high school or higher), marital status (single/widowed/divorced vs. married), presence of hypertension, diabetes, dyslipidemia, obesity, and family history of CVD (not present vs. present). Additionally, both anxious and depressive symptoms and the season during which data were collected for each participant were added as time-varying covariates at level 1 of the H(G)LM models. We further assessed and reported pseudo-R^2^, a measure of HLMs’ effect sizes indicating the proportion of within-person variance accounted for by adding the linear parameters [31]. All multilevel models and further information on piecewise regressions are reported in the Appendix A.

Analyses were performed using the Statistical Package for Social Sciences (SPSS) version 26.0 and Hierarchical Linear Models (HLM) Professional version 8.0. All statistical tests were two-tailed, and a *p* ≤ 0.05 was considered statistically significant. 

## 3. Results

### 3.1. Study Population

The study population included 275 consecutive ACS patients at their first coronary event, aged 57.1 ± 7.87 years; 84% were men and 16% women. The proportion of men in the sample was a direct consequence of the incidence of ACS, which is more common among men than women [34]. The patients’ mean BMI was 27.2 ± 4.1 kg/m^2^; their mean waist circumference [WC] was 96.5 ± 11.1 cm. All of them were Caucasian. Almost all patients were prescribed pharmacological treatment for ACS, consisting of antiplatelet drugs (99% of patients), beta-blockers (89%), statins (97%), sartans, or ace-inhibitors (99%). Further demographic and clinical variables are reported in Table 1 and Table 2.

Regarding the psychological assessment, the results showed that, on average, patients were not affected by clinically significant anxiety (the mean score overtime was 5.78, with a cut-off of 7), or clinically significant depression (the mean score overtime was 3.16, with a cut-off of 7). Table 3 reports a detailed description of anxiety and depression scores across all time points. 

Regarding drop-outs, 12.7% of patients were absent at the six-month follow-up, 14.9% at the one-year, 20.7% at the two-year, 33.1% at the three-year, and 35.3% at the five-year. In comparison, percentages of drop-outs at the one-year follow-up were similar to those reported in other European studies on ACS patients [35]. Causes of drop-out included: loss to follow-up, relocation, refusal, and, in a small minority of cases, inability to track down the patient. It is worth noting that this study’s statistical techniques enabled us to use all the data available and not only those provided by completers. Therefore, the final number of participants remained 275.

### 3.2. Longitudinal Changes in Protein Intake

Preliminary analyses showed that both the time slopes (Time.D1 and Time.D2; see Appendix A for the multilevel model) for the variables Fish and Legume intake were non-significant and were therefore fixed in subsequent analyses. Similarly, the pre-to-post event time slope for the variable red/processed meat intake was non-significant, and its effect was treated as fixed. In both cases, this suggested that participants had similar changes over time in the aforementioned eating behaviors (i.e., the slopes were not significantly different between participants).

As regards fish intake, results showed that before the onset of cardiovascular disease (i.e., at the intercept), 66.4% of patients consumed two or more servings of fish per week. From pre-event to six months later (i.e., the “Time.D1” slope β1), patients increased their fish intake. Those who had a family history of CVD had a greater increase in fish intake (β = 1.31; *p* = 0.043) than those without a family history of CVD. A significant longitudinal decrease in fish intake was found from the six-month to the five-year follow-up (i.e., the “Time.D2” slope β2). Anxiety, depression, and seasonal variations were not significant predictors.

As regards legume intake, results showed that before the onset of CVD (i.e., at the intercept), a high percentage (79.1%) of patients consumed two or more servings of legumes per week. Older patients reported higher levels of legume intake (β = 0.07; *p* = 0.007). From pre-event to six months after (i.e., the “Time.D1” slope β1), patients maintained stable levels of legume intake, except older patients, who decreased their consumption of legumes (β = −0.13; *p* = 0.009). A significant longitudinal decrease in legume intake was found from the six-month to the five-year follow-up (i.e., the “Time.D2” slope β2). This decrease was smaller among patients affected by hypertension (β = 0.15; *p* = 0.035), and dyslipidemia (β = 0.15; *p* = 0.041). Anxiety, depression, and seasonal variations were not significant predictors.

Regarding the consumption of red/processed meat, results showed that before the onset of cardiovascular disease (i.e., at the intercept), only 8.4% of patients consumed two or fewer servings of red/processed meat per week. From pre-event to six months after (i.e., the “Time.D1” slope β1), red meat consumption significantly declined; indeed, 33.5% of patients consumed two or fewer servings of red/processed meat per week. This decrease was greater among patients affected by hypertension (β = 1.87; *p* = 0.031). From the six-month to the five-year follow-up (i.e., the “Time.D2” slope β2), a significant longitudinal decrease in red/processed meat consumption was found. Patients who were married decreased their consumption of red/processed meat more than patients who were single, widowed, or divorced (β = 0.20; *p* = 0.041). Patients affected by hypertension decreased their consumption of red/processed meat less than patients not affected by hypertension (β = −0.24; *p* = 0.004). Patients with higher levels of anxiety decreased their consumption of red/processed meat more than patients with lower anxiety levels (β = 0.09; *p* = 0.025). There was no statistically significant effect of depression on red/processed meat consumption. The decrease in red/processed meat consumption was significantly higher in spring (β = 0.47; *p* = 0.016) and in autumn (β = 0.43; *p* = 0.041) than in winter. Further, the consumption of red/processed meat was significantly lower in spring than in summer (β = 0.48; *p* = 0.027). The lowest consumption of red/processed meat was recorded in spring (frequency = 165, 39.3% of patients ate a healthy amount of meat); it increased in summer (frequency = 75, 28.8%), decreased in autumn (frequency = 120, 39.2%), and increased again in winter (frequency = 115, 34%).

### 3.3. Longitudinal Changes in Physical Activity

Regarding physical activity, results showed that before the onset of cardiovascular disease (i.e., at the intercept), patients affected by hypertension performed less physical activity (β = −0.63; *p* = 0.011) compared to patients not affected by hypertension. From pre-event to six months after (i.e., the “Time.D1” slope β1), patients increased their physical activity. Patients with higher educational levels had steeper increases (β = 0.99; *p* = 0.036) than those with less education. Patients affected by hypertension increased their levels of physical activity (β = 1.08; *p* = 0.025). A significant longitudinal decrease in levels of physical activity was found from the six-month to the five-year follow-up (i.e., the “Time.D2” slope β2). Patients who were married decreased their physical activity levels more than patients who were single, widowed, or divorced (β = −0.12; *p* = 0.048). Anxiety and depression were not significant predictors. Physical activity was significantly higher in autumn than in winter (β = 0.31; *p* = 0.015). The difference between the amount of physical activity in summer and winter approached significance (β = 0.28; *p* = 0.048). The highest amount of physical activity was recorded in autumn (mean = 5.11 ± 1.88); it decreased significantly in winter (mean = 4.65 ± 2.09), then rose in a variable manner in spring (mean = 4.92 ± 1.93) and summer (mean = 4.83 ± 2.06).

**Figure 1:**
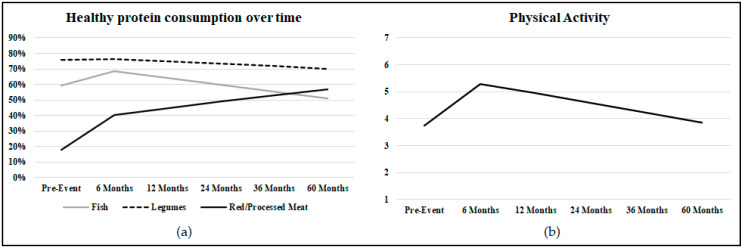
(**a**) The graph shows the longitudinal trajectory of healthy protein consumption (i.e., two or more portions of fish per week—grey line, two or more portions of legumes per week—dashed line, two or fewer portions of red meat per week/processed—black line). The time is represented on the horizontal axis, the percentage of patients showing healthy behavior is represented on the vertical axis; (**b**) the graph shows the longitudinal trajectory of the amount of physical activity. The time is represented on the horizontal axis; the average physical activity score is represented on the vertical axis. The total score ranges from 1 to 7, with higher scores indicating a healthier amount of physical activity. Scores higher than 6 indicate the target amount of physical activity for cardiovascular prevention.

Frequencies, percentages, means, and standard deviations for all outcome variables across all time points are reported in Table 4. All regression coefficients, standard errors, t and *p* values for β1 and β2 slope parameters are reported in Table 5. All fixed effects are reported in the Supplementary Materials. Figure 1 illustrates the longitudinal trajectory of healthy protein consumption and the amount of physical activity.

## 4. Discussion

The present study explored the longitudinal trajectories of physical activity and protein consumption in a cohort of patients with ACS at their first event. The aim was to assess whether and which factors could play a significant role in predicting healthier behavior. For the first time, this study has investigated the role played by anxiety and depressive symptoms, and the change of seasons.

Our results are in agreement with previous studies showing that most patients affected by CVD fail to achieve healthy lifestyle targets [11,13,36,37]. They showed that, on average, patients experienced significant increases in the levels of healthy behaviors from pre- to post-cardiovascular event. However, from post-event to the five-year follow-up, patients showed a significant decline in their levels of healthy behaviors. This demonstrates the difficulty of patients not so much in assuming but maintaining the recommended healthy behaviors in the long term. In particular, as regards fish intake, results showed that a good percentage of patients consumed two or more servings of fish per week before the onset of the ACS, which has been related to positive health outcomes [27]. Immediately after the cardiovascular event, fish consumption increased, especially among patients with a family history of CVD. We could argue that patients with this risk factor received more health-related suggestions or were more sensitive to prescriptions received regarding fish intake, possibly due to a greater risk perception than patients with no family history of CVD. In support of this hypothesis, a cross-sectional study showed that a family history of CVD was associated with both higher risk perception and the adoption of a healthier lifestyle in people diagnosed with familial hypercholesterolemia [38]. Overall, the findings therefore suggest that healthcare professionals should be aware that some people may underestimate the risk of CVD and emphasize how behavior change can reduce risk [38]. This consideration is reinforced by the evidence that adherence declined over time in our sample, highlighting a great difficulty in maintaining positive behavior.

Similar results were obtained in the case of legume intake. Indeed, before the onset of the ACS, many patients consumed the recommended two or more servings of legumes per week [27], especially older adults. Immediately after the cardiovascular event, patients maintained stable levels of legume intake. Considering the high percentage of patients who consumed a quantity of legumes in line with recommendations before the cardiac event, it is not surprising that the behavior was maintained and did not increase further. This observation is reinforced by the fact that older patients decreased legume intake, regressing towards the mean. Over the five-year follow-up, legume consumption decreased, except for patients affected by hypertension and dyslipidemia. Again, the presence of multiple risk factors could be associated with higher risk perception. Previous studies have shown that reporting a high perceived CVD risk in the general population was strongly associated with treatment for hypertension, diabetes, or dyslipidemia [39]. This could make patients more alert and receptive to physicians’ prescriptions during cardiovascular rehabilitation and have an effect, albeit weak, in the long term. Again, this finding underlines that healthcare professionals should be aware that some patients underestimate the risks associated with CVD and are therefore less likely to change their health-related behavior stably.

As regards the consumption of red and processed meat, results showed that a small percentage of patients consumed the recommended two or fewer servings of red and processed meat per week before the cardiovascular event [27]. Immediately after the cardiovascular event, patients significantly decreased red and processed meat intake. Consumption continued to decrease over time, especially among patients who were married. This result goes in the same direction as studies that showed this demographic variable’s protective role in fostering a healthier diet [13,40], and it emphasizes the importance that the partner can have in motivating and supporting the patient to adopt a healthy diet. A second relevant observation is that immediately after the cardiovascular event, patients with hypertension adopted healthier behavior than patients without this risk factor. However, after the first follow-up, they decreased their red and processed meat intake less than patients without this risk factor, regressing towards the mean. This result underlines once again how the presence of multiple risk factors can make patients more alert and willing to follow medical indications to adopt a healthier lifestyle, although there is a difficulty in maintaining healthy behavior stably.

Interestingly, patients with higher anxiety levels decreased red and processed meat intake more than patients with lower levels of anxiety over the five-year follow-up. Among psychological disorders, anxiety, together with depression, is the most widely and frequently studied condition. Anxiety has been linked to health status and several risk factors predisposing CVD [41]. Previous studies have reported contrasting results of the impact of anxiety on manifold health-related outcomes [42,43]. In line with our findings, Grace and colleagues [14] found that patients with anxiety symptoms were more likely to participate in cardiovascular rehabilitation. Moreover, Parker and colleagues [44] found that patients who had been hospitalized for an ACS and suffered from generalized anxiety disorder had a superior five-year outcome than patients with different psychological symptoms. The group discussed the positive contribution of worry in medication adherence and a proactive approach to health practitioners. However, to the best of our knowledge, no studies have explored if a relatively high level of anxiety could play a role in promoting a positive long-term lifestyle change, specifically in adopting a healthier diet. Feeling “tense or wound up”, getting “a sort of frightened feeling as if something awful were about to happen”, experiencing “worrying thoughts” about one’s health can somehow promote awareness of the risks to one’s health and consequently motivate one to change one’s behavior. Consistently, a study focused on sedentary young adults [45] found that the exposure to web-based images and information about cardiovascular risk could lead to higher levels of worry and risk perception, contributing to increase motivation in adopting a healthier behavior. Our findings suggest that cardiovascular rehabilitation programs would benefit from the introduction of psychological interventions aimed, on the one hand, at treating the emotional aspects related to anxiety, providing psychological support, and promoting coping [14,41], and on the other hand, at exploiting the concern patients express about their health to promote cognitive awareness of the risks associated to CVD recurrence. This awareness could sustain patients’ abilities to self-regulate their behaviors and increase their beliefs about their self-efficacy [46,47,48]. Self-efficacy refers to the belief in one’s capabilities to organize and execute the actions required to produce given attainments [49]. Such beliefs are particularly relevant with regard to health-related behaviors [50]. In the context of CVD, it has been shown that self-efficacy plays a crucial role in adopting and maintaining a healthy diet [26,51] and a physically active lifestyle [51,52]. Especially for maintenance, the confidence in one’s abilities to preserve the newly developed behavior in spite of the possible barriers is pivotal. Indeed, as already suggested: “once an action has been taken, individuals with high maintenance self-efficacy invest more effort and persist longer than those who are less self-efficacious” [53]. Regarding depression, our results go in the same direction as a previous study in which we found that higher levels of depression at baseline were not associated with a subsequent unhealthy lifestyle six or twelve months later [19]. It should be noted that only a very small percentage of patients experienced anxiety and depressive symptoms, so it was probably not possible to capture any effects of this psychological variable.

The last observation regarding the longitudinal trajectory in red and processed meat consumption is that it was affected by seasonal variations. Red and processed meat intake was significantly lower in spring and autumn than in winter and summer. This result is not surprising if we consider the seasonal cultural habits of the Mediterranean area, where the patients of this study come from. Indeed, winter, especially Christmastime, and summer are the times when the longest holidays are taken in Italy. It is possible that it is more difficult to follow a healthy diet, particularly at these times. Similar trends have been described in a recent meta-analysis [21], suggesting a significant decrease of meat consumption from summer to autumn across different European countries. Interestingly, those food groups characterized by less healthy consumption (high consumption of unhealthy foods, or low consumption of healthy foods) were also characterized by consumption that varied with the season [22]. Consistent with this observation, in the present study, the role of seasonality emerged on the consumption of red and processed meat, whose intake was on average less healthy than the fish and legume intake. Maintaining a low consumption of red and processed meat was the eating behavior that saw the greatest effort, and in which the greatest change was observed. Perhaps for this reason, it is the behavior where a greater sensitivity to exceptions emerges. It is possible that patients were led to allow themselves exceptions, being more sensitive to contextual factors linked to the change in seasons in the case of red and processed meat consumption than in fish and legume consumption. This trend highlights, once again, the need for tailored cardiovascular rehabilitation programs. Patients’ preference for red/processed meat intake may differ between seasons, and this variation may impact adherence to a healthy lifestyle. Patients should be aware that the incidence of CVDs has seasonal peaks [23], and the incidence of cardiovascular events could be, at least in part, associated with the difficulties they may encounter at different seasons of the year in adopting a healthy lifestyle. Patients should be supported in finding strategies that can compensate for variations in healthy behavior linked to contextual factors and seasonal variations (i.e., weather, holiday periods, or vice versa of greater workload). It is useful to underline once again that red/processed meat is the protein source for which the unhealthier behavior was recorded before the cardiovascular event and for which the most significant changes in consumption were observed over time; perhaps for this reason, among the protein sources examined in the present study, it is also the one in which the role of several predictors emerged.

Finally, as regards physical activity, the results of the present study showed that patients reported sub-optimal amounts of physical activity at each time-point. Accordingly, previous studies showed that adherence to good physical activity levels is one of the most difficult goals to reach and maintain among patients with CVDs [11,12,46]. Before the onset of cardiovascular disease, patients with hypertension were less physically active than those without hypertension. This observation is consistent with previous studies [54], and it is relevant considering that regular exercise is one of the most important activities for primary prevention of hypertension and improving long-term survival [54]. Immediately after the cardiovascular event, patients significantly increased their physical activity. This increase was higher among patients with hypertension, and those with higher educational levels. This last result goes in the same direction as studies that have shown that this demographic variable plays a protective role [55,56]. Low levels of education have been associated with low levels of health literacy [55]. Health literacy is defined as the set of skills to access, understand, and evaluate information to make health related decisions in everyday life. Poor health literacy has been linked to less healthy behaviors. Our results support, albeit indirectly, studies that emphasize the crucial role of health literacy in the success of cardiovascular rehabilitation programs [55]. After the first time-point, a significant longitudinal decrease in physical activity levels was found, decreeing a failure to adopt a healthier behavior stably. Patients who were married decreased their physical activity levels more than patients who were single, widowed, or divorced. This result may seem surprising when we generally consider being married as an indicator of social support. For red and processed meat consumption, we found the opposite pattern. This difference could lie in the fact that in the sample of the present study, made up mostly of men in their 60s, the wife represents the partner. In this generation and the Mediterranean area culture, it is common for women to cook, so we may infer that somehow their presence promotes a healthier diet [13,40]. In the case of physical activity, patients must actively engage themselves in doing more physical activity. It is also possible that partners hinder rather than encourage this behavior, which leads patients to choose between exercise time and family time. Indeed, our results may suggest that patient caregivers should also be included in the physical activity education programs. It is also important to underline that it is not so much being married (an indicator of structural social support), but rather the quality of the relationship (the so-called functional social support) that plays a protective role in the behavior of adherence to the prescriptions for one’s health [57]. Therefore the “marital status” variable can only capture part of the phenomenon linked to social support.

The highest amount of physical activity was recorded in autumn; it decreased significantly in winter, and rose variably in spring and summer. These results show that patients tend to be more active after the summer; in winter, perhaps due to the cold, they have more difficulty maintaining a sufficient physical activity level. In spring, probably being able to resume physical activity outdoors, they become more active again, but with the heat of summer, the trend goes down once more. The seasonal variation of recreational physical activity has been reported in several studies [20]. In the CVD population, it has been argued that the decrease in physical activity from summer to winter was mainly due to an increase in symptom severity in winter, making exercise difficult to maintain over all the seasons [25]. Nevertheless, seasonal variations have seldom been considered a factor that modulates compliance with healthy regimens in this population [24,25]. Indeed, prior literature has also suggested taking seasonal differences in physical activity levels into consideration in future research, in order to better understand their influence on long-term adherence [58]. As discussed above, our findings suggest the need to pay attention to the role of environmental variations in CVD patients’ adherence to healthy lifestyles, and they confirm the need for tailored cardiovascular rehabilitation programs that help patients overcome the difficulties they may face in adopting a healthy lifestyle in different seasons of the year. For example, at specific times of the year, it would be useful to suggest alternative activities, if those preferred by patients are outdoors (walking).

The present study has a series of limitations. First, despite using the piecewise regression approach, which allows for modeling the slope discontinuity from pre-event to six months after, information about diet and physical activity before the onset of the ACS was measured retrospectively by asking patients to report their habits before the acute event. This approach may limit the results’ reliability, because patients may have over- or underestimated their real past healthy lifestyle. Second, both diet and physical activity were evaluated through self-report measures and not through an objective behavioral measure. Nevertheless, this methodology is widely adopted in the medical and psychological literature [26,27,28], and it has been proven to correlate with more objective measurement methods [59].

Despite these limitations, the present study reports several original findings and has important implications for behavioral intervention in cardiovascular disease patients. First, patients’ protein consumption and physical activity were evaluated over a long period of time. Second, an advanced statistical technique (HLMs) with a piecewise regression approach was used to model the individual change rate from pre-event to five-year post-cardiovascular event. This technique allowed us to test the hypotheses that patients experienced both increases and decreases in healthy protein consumption and physical activity in subsequent periods of time. Overall, this approach allowed us to demonstrate that patients’ main difficulty was not in assuming but in maintaining the recommended healthy behaviors in the long term. Third, the study focused on a homogeneous sample of patients at their first cardiovascular event. While the homogeneity of the sample may limit the generalizability of the results, it ensures that the inferences drawn from the study apply effectively to the population of ACS patients at their first cardiac event. Therefore, they assume an important significance for secondary cardiovascular prevention. Finally, by simultaneously considering several predictors of healthy behavior, our results confirmed the protective role of specific sociodemographic and clinical characteristics. For the first time, the roles of both environmental and psychological factors have been considered.

## 5. Conclusions

In summary, the present study shows that patients with ACS experience difficulties in achieving and mostly maintaining adequate levels of a healthy diet and physical activity over time. These difficulties are modulated by environmental conditions, and most importantly, by psychological characteristics. These findings suggest how to tailor diet and physical activity interventions. Tailoring should consider that patients’ preference for diet and physical activity could differ between seasons, which could impact their adherence to a healthy lifestyle. Tailoring should also be aimed at promoting cognitive awareness of the risks associated with CVD recurrence. In fact, our study results showed that patients who were more anxious, therefore more concerned and somehow aware of their health, were more able to maintain healthy behavior over time. Cognitive awareness of the risks associated with CVD recurrence may be a useful tool to sustain patients’ capabilities to self-regulate their behaviors and to ameliorate lifestyle behavior. 

## Figures and Tables

**Table 1:** Sociodemographic characteristics of the sample (*N* = 275). Means and standard deviations (SD) are reported for age. Frequencies (*n*) and percentages are reported for gender, working status, educational level, and marital status.

Sociodemographic Variables	ACS Patients
Age, mean (SD)	57.1 (7.87)
Gender, *n* (%)	
Male	231 (84%)
Female	44 (16%)
Working status, *n* (%)	
working	111 (40.4%)
not working	163 (59.3%)
Educational level, *n* (%)	
less than high school	141 (51.3%)
high school or higher	134 (48.7%)
Marital status, *n* (%)	
single\widowed\divorced	78 (28.4%)
married	197 (71.6%)

**Table 2:** Clinical characteristics of the sample (*N* = 275). Frequencies (*n*) and percentages are reported for clinical presentation, percutaneous coronary intervention, patients with at least one stent, and risk factors. Means and standard deviations (SD) are reported for body mass index, systolic blood pressure, and diastolic blood pressure.

Clinical Variables	ACS Patients
Clinical Presentation, *n* (%)	
Non-ST elevation myocardial infarction (NSTEMI)	54 (19.8)
ST-elevation myocardial infarction (STEMI)	196 (71.8)
Unstable Angina	23 (8.5)
Percutaneous coronary intervention, *n* (%)	258 (94.5)
Patients with at least one stent, *n* (%)	263 (96)
Risk factors, *n* (%)	
Hypertension	127 (46.5)
Dyslipidemia	143 (52.4)
Smoking History	180 (66.4)
Diabetes	47 (17.2)
Obesity	43 (15.8)
Family History of CVD	108 (39.3)
Physical Inactivity	20 (7.3)
Body Mass Index, mean (SD)	27.2 (4.1)
Systolic Blood Pressure (SBP), mean (SD)	115.9 (13.9)
Diastolic Blood Pressure (DBP), mean (SD)	72.9 (8.5)

**Table 3:** Sample size (N) and percentages of patients with a mean score above the cut-off of 7 for anxiety and depression during all time points (baseline, six-month, one-, two-, three- and five-year follow-up). The cut-off of 7 defines the presence of clinically significant symptoms of anxiety and depression. Means and standard deviations (SD) of the sample for each time point are also reported.

	Baseline		6 Months		1 Year		2 Years		3 Years		5 Years	
	*N* (%)	Mean (SD)	*N* (%)	Mean (SD)	*N* (%)	Mean (SD)	*N* (%)	Mean (SD)	*N* (%)	Mean (SD)	*N* (%)	Mean (SD)
Anxiety	274 39%	6.85 (3.88)	241 33%	6.23 (3.35)	233 26%	5.70 (3.45)	218 28%	5.99 (3.37)	183 26%	5.68 (3.34)	175 14%	4.21 (3.19)
Depression	274 21%	4.73 (3.42)	241 19%	4.45 (3.18)	233 11%	3.84 (2.98)	218 17%	4.45 (3.14)	183 15%	4.25 (3.29)	175 14%	2.90 (2.94)

**Table 4:** Descriptive statistics of the outcome measures at all time points (pre-event, six-month, one-, two-, three- and five-year follow-up). Frequencies (*n*) and percentages are reported for the protein consumption outcomes (fish, legumes, and healthy consumption of red/processed meat). Means and standard deviations (SD) are reported for physical activity.

	**Pre**		**6 Months**		**1 Year**		**2 Years**		**3 Years**		**5 Years**	
	**N**	***n* (%)**	**N**	***n* (%)**	**N**	***n* (%)**	**N**	***n* (%)**	**N**	***n* (%)**	**N**	***n* (%)**
Fish	274	182 (66.4)	239	185 (77.4)	233	186 (67.6)	216	124 (57.4)	183	105 (57.4)	176	109 (61.9)
Legumes	273	216 (79.1)	238	195 (81.9)	231	190 (82.3)	218	151 (69.3)	183	124 (67.8)	175	131 (74.9)
Red/processed meat	274	23 (8.4)	239	80 (33.5)	233	75 (32.2)	218	105 (48.2)	184	85 (46.2)	176	107 (60.8)
	**N**	**Mean (SD)**	**N**	**Mean (SD)**	**N**	**Mean (SD)**	**N**	**Mean (SD)**	**N**	**Mean (SD)**	**N**	**Mean (SD)**
Physical Activity	275	4.20 (2.02)	240	5.48 (1.79)	234	5.36 (1.80)	217	5.17 (1.89)	183	5.12 (1.94)	176	3.86 (1.96)

Data on lifestyle behaviors at pre-event were collected retrospectively once patients were hospitalized.

**Table 5:** Fixed effects for the longitudinal changes in behavioral outcomes (fish intake, legume intake, red/processed meat intake, physical activity).

Variables	Fixed Effects										
	β_1_	SE	t Values	DF	*p*	β_2_	SE	t Values	DF	*p*	R^2^
Fish intake	0.902	0.324	2.783	1006	0.005	−0.213	0.040	−5.341	1006	<0.001	\
Legume intake	−0.035	0.339	−0.103	1003	0.918	−0.085	0.038	−2.240	1003	0.025	\
Red/processed meat intake	4.011	0.508	7.895	749	<0.001	0.276	0.044	6.351	260	<0.001	\
Physical Activity	2.334	0.229	10.217	260	<0.001	−0.240	0.025	−9.623	260	<0.001	34.4%

*β*_1_ = unstandardized regression coefficient for the average growth rate from pre-event to six months after. *β*_2_ = unstandardized regression coefficient for the average growth rate from the six-month follow-up to the five-year follow-up. SE = standard error of the regression coefficient; DF = degrees of freedom (df changes due to missing values or to the presence of fixed\random time slopes). R^2^ refers to pseudo-R^2^, indicating the proportion of within-person variance accounted for by adding the “Time.1” and “Time.2” parameter to the model; it cannot be analyzed for dichotomous variables.

## Data Availability

The data presented in this study are available on request from the corresponding author. The data are not publicly available due to privacy and ethical restrictions.

## Supplementary Materials

The following are available online at https://www.mdpi.com/2072-6643/13/2/634/s1: Table S1: Fixed effects (with robust standard errors) for the dependent variable “Physical Activity” (MDS8); Table S2: Fixed effects (with robust standard errors) for the dependent variable “Legume intake”; Table S3: Fixed effects (with robust standard errors) for the dependent variable “Fish Intake”; Table S4: Fixed effects (with robust standard errors) for the dependent variable “Red/processed Meat Intake”.

## Appendix A

**HGLM 2-level models 1**

*Level-1 Model*

Prob(Y*_ti_* = 1|*π_i_*) = *ϕ_ti_*

log[*ϕ_ti_*/(1 − *ϕ_ti_*)] = η*_ti_*

η_ti_ = π_0i_ + π_1i_ * (TIME.1_ti_) + π_2i_ * (TIME.2_ti_) + π_3i_ * (HADS.Anxiety_ti_) + π_4i_ * (HADS.Depression_ti_) + π_5i_ * (Season.Spring_ti_) + π_6i_ * (Season.Summer_ti_) + π_7i_ * (Season.Autumn_ti_) + e_ti_

*Level-2 Model*

π_0i_ = β_00_ + β_01_ * (AGE_i_) + β_02_ * (SEX_i_) + β_03_ * (WORKING_STATUS_i_) + β_04_ * (MARITAL_STATUS_i_) + β_05_ * (EDUCATION_i_) + β_06_ * (LIVING_STATUS_i_) + β_07_ * (FAMILY_HISTORY_i_) + β_08_ * (HYPERTENSION_i_) + β_09_ * (DIABETES_i_) + β_010_ * (DYSLIPIDEMIA_i_) + β_011_ * (OBESITY_i_) + r_0i_

π_1i_ = β_10_ + β_11_ * (AGE_i_) + β_12_ * (SEX_i_) + β_13_ * (WORKING_STATUS_i_) + β_14_ * (MARITAL_STATUS_i_) + β_15_ * (EDUCATION_i_) + β_16_ * (LIVING_STATUS_i_) + β_17_ * (FAMILY_HISTORY_i_) + β_18_ * (HYPERTENSION_i_) + β_19_ * (DIABETES_i_) + β_110_ * (DYSLIPIDEMIA_i_) + β_111_ * (OBESITY_i_) + r_1i_

π_2i_ = β_20_ + β_21_ * (AGE_i_) + β_22_ * (SEX_i_) + β_23_ * (WORKING_STATUS_i_) + β_24_ * (MARITAL_STATUS_i_) + β_25_ * (EDUCATION_i_) + β_26_ * (LIVING_STATUS_i_) + β_27_ * (FAMILY_HISTORY_i_) + β_28_ * (HYPERTENSION_i_) + β_29_ * (DIABETES_i_) + β_210_ * (DYSLIPIDEMIA_i_) + β_211_ * (OBESITY_i_) + r_2i_

π_3i_ = β_30_

π_4i_ = β_40_

π_5i_ = β_50_

π_6i_ = β_60_

π_7i_ = β_70_

*Note.* Y = Dependent Variable (Fish intake, Legume intake, and Red/processed meat intake, dichotomized); The level-1 dummy-coded variable “Season” was uncentered, while HADS Anxiety and Depression were group-mean centered. All level-2 variables were centered around the grand mean.

**HLM 2-level models 2**

*Level-1 Model*

Y_ti_ = π_0i_ + π_1i_ * (TIME.1_ti_) + π_2i_ * (TIME.2_ti_) + π_3i_ * (HADS.Anxiety_ti_) + π_4i_ * (HADS.Depression_ti_) + π_5i_ * (Season.Spring_ti_) + π_6i_ * (Season.Summer_ti_) + π_7i_ * (Season.Autumn_ti_) + e_ti_

*Level-2 Model*

π_0i_ = β_00_ + β_01_ * (AGE_i_) + β_02_ * (SEX_i_) + β_03_ * (WORKING_STATUS_i_) + β_04_ * (MARITAL_STATUS_i_) + β_05_ * (EDUCATION_i_) + β_06_ * (LIVING_STATUS_i_) + β_07_ * (FAMILY_HISTORY_i_) + β_08_ * (HYPERTENSION_i_) + β_09_ * (DIABETES_i_) + β_010_ * (DYSLIPIDEMIA_i_) + β_011_ * (OBESITY_i_) + r_0i_

π_1i_ = β_10_ + β_11_ * (AGE_i_) + β_12_ * (SEX_i_) + β_13_ * (WORKING_STATUS_i_) + β_14_ * (MARITAL_STATUS_i_) + β_15_ * (EDUCATION_i_) + β_16_ * (LIVING_STATUS_i_) + β_17_ * (FAMILY_HISTORY_i_) + β_18_ * (HYPERTENSION_i_) + β_19_ * (DIABETES_i_) + β_110_ * (DYSLIPIDEMIA_i_) + β_111_ * (OBESITY_i_) + r_1i_

π_2i_ = β_20_ + β_21_ * (AGE_i_) + β_22_ * (SEX_i_) + β_23_ * (WORKING_STATUS_i_) + β_24_ * (MARITAL_STATUS_i_) + β_25_ * (EDUCATION_i_) + β_26_ * (LIVING_STATUS_i_) + β_27_ * (FAMILY_HISTORY_i_) + β_28_ * (HYPERTENSION_i_) + β_29_ * (DIABETES_i_) + β_210_ * (DYSLIPIDEMIA_i_) + β_211_ * (OBESITY_i_) + r_2i_

π_3i_ = β_30_

π_4i_ = β_40_

π_5i_ = β_50_

π_6i_ = β_60_

π_7i_ = β_70_

*Note.* Y = Dependent Variable (Physical Activity, continuous); The level-1 dummy-coded variable “Season” was uncentered, while HADS Anxiety and Depression were group-mean centered. All level-2 variables were centered around the grand mean.
