# Supplementary material for: Protein Intake and Physical Activity in Newly Diagnosed Patients with Acute Coronary Syndrome: A 5-Year Longitudinal Study
Source: Nutrients. 2021 Feb 16;13(2):634. doi: 10.3390/nu13020634 (PMC7919823; doi:10.3390/nu13020634)
Supplement: Supplementary file 1 [file nutrients-13-00634-s001.pdf]

Supplementary Table S1.

Fixed effects (with robust standard errors) for the dependent variable “Physical Activity” (MDS8).

**Fixed Effects for MDS8**	**Coefficient**	**SE**	***t*-ratio**	**DF**	***p*-value**
**Intercept (Pre-Event)**					
Intercept	3.992206	0.147342	27.095	260	<0.001
Age	0.018782	0.018072	1.039	260	0.300
Sex	-0.368825	0.313151	-1.178	260	0.240
Working Status	-0.255810	0.296150	-0.864	260	0.389
Marital Status	0.221033	0.287656	0.768	260	0.443
Education	-0.032569	0.244483	-0.133	260	0.894
Hypertension	-0.633864	0.246909	-2.567	260	0.011
Diabetes	-0.213147	0.332258	-0.642	260	0.522
Dyslipidaemia	0.081775	0.249780	0.327	260	0.744
Obesity	-0.133832	0.354109	-0.378	260	0.706
Family History of CVD	-0.017764	0.249603	-0.071	260	0.943
**Slope Time.1 (Pre- to Post-Event)**					
Intercept	2.337891	0.228819	10.217	260	<0.001
Age	-0.041171	0.033366	-1.234	260	0.218
Sex	-0.716761	0.621525	-1.153	260	0.250
Working Status	0.289097	0.568911	0.508	260	0.612
Marital Status	0.311788	0.572042	0.545	260	0.586
Education	0.985863	0.468671	2.104	260	0.036
Hypertension	1.076834	0.479193	2.247	260	0.025
Diabetes	0.020635	0.640066	0.032	260	0.974
Dyslipidaemia	-0.255119	0.459711	-0.555	260	0.579
Obesity	-0.241187	0.643538	-0.375	260	0.708
Family History of CVD	0.358404	0.458113	0.782	260	0.435
**Slope Time.2 (Post- to 5-years follow-up)**					
Intercept	-0.240320	0.024975	-9.623	260	<0.001
Age	-0.000211	0.003628	-0.058	260	0.954
Sex	0.113187	0.073177	1.547	260	0.123
Working Status	0.066401	0.060602	1.096	260	0.274
Marital Status	-0.116579	0.058561	-1.991	260	0.048
Education	0.019499	0.051501	0.379	260	0.705
Hypertension	-0.040100	0.050029	-0.802	260	0.424
Diabetes	-0.051105	0.065714	-0.778	260	0.437
Dyslipidaemia	-0.020676	0.049280	-0.420	260	0.675
Obesity	0.001137	0.074348	0.015	260	0.988
Family History of CVD	-0.041331	0.052463	-0.788	260	0.432
**Slope for HADS Anxiety**					
Intercept	-0.005129	0.025535	-0.201	489	0.841

**Slope for HADS Depression**					
Intercept	-0.043894	0.027452	-1.599	489	0.110
**Slope for Spring (reference category: Winter)**					
Intercept	0.224986	0.134143	1.677	489	0.094
**Slope for Summer (reference category: Winter)**					
Intercept	0.275773	0.142972	1.929	489	0.054
**Slope for Autumn (reference category: Winter)**					
Intercept	0.305470	0.124998	2.444	489	0.015

Note. Coefficient = unstandardized regression coefficient. SE = standard error of the regression coefficient; DF = degrees of freedom (df changes due to missing values or to the presence of fixed\random time slopes).

Supplementary Table S2.

Fixed effects (with robust standard errors) for the dependent variable "Legume Intake".

Fixed Effects for Legumes intake	Coefficient	SE	*t*-ratio	DF	*p*-value
**Intercept (Pre-Event)**					
Intercept	1.626591	0.246810	6.590	260	<0.001
Age	0.073220	0.026796	2.733	260	0.007
Sex	-0.328878	0.465244	-0.707	260	0.480
Working Status	-0.301787	0.438684	-0.688	260	0.492
Marital Status	0.501588	0.384423	1.305	260	0.193
Education	0.331629	0.353101	0.939	260	0.349
Hypertension	-0.242478	0.359984	-0.674	260	0.501
Diabetes	0.033605	0.464234	0.072	260	0.942
Dyslipidaemia	-0.208812	0.345973	-0.604	260	0.547
Obesity	-0.041069	0.481286	-0.085	260	0.932
Family History of CVD	-0.007595	0.355497	-0.021	260	0.983
**Slope Time.1 (Pre- to Post-Event)**					
Intercept	-0.034833	0.338560	-0.103	1003	0.918
Age	-0.128034	0.048749	-2.626	1003	0.009
Sex	-0.102963	0.795026	-0.130	1003	0.897
Working Status	0.544923	0.869119	0.627	1003	0.531
Marital Status	-0.116674	0.758195	-0.154	1003	0.878
Education	-0.373167	0.682938	-0.546	1003	0.585
Hypertension	-0.379641	0.699042	-0.543	1003	0.587
Diabetes	0.226964	0.864289	0.263	1003	0.793
Dyslipidaemia	-0.039697	0.686289	-0.058	1003	0.954
Obesity	-0.414248	0.899825	-0.460	1003	0.645
Family History of CVD	-0.079542	0.684819	-0.116	1003	0.908
**Slope Time.2 (Post- to 5-years follow-up)**					
Intercept	-0.085059	0.037979	-2.240	1003	0.025
Age	0.008147	0.005872	1.387	1003	0.166
Sex	-0.054821	0.078948	-0.694	1003	0.488
Working Status	-0.099882	0.099758	-1.001	1003	0.317
Marital Status	0.006935	0.079506	0.087	1003	0.931
Education	0.089071	0.071560	1.245	1003	0.214
Hypertension	0.152142	0.072076	2.111	1003	0.035
Diabetes	-0.137396	0.107256	-1.281	1003	0.200
Dyslipidaemia	0.148356	0.072507	2.046	1003	0.041
Obesity	0.000008	0.080543	0.000	1003	1.000
Family History of CVD	-0.056686	0.069321	-0.818	1003	0.414
**Slope for HADS Anxiety**					
Intercept	0.060941	0.032202	1.892	1003	0.059

**Slope for HADS Depression**					
Intercept	-0.047894	0.036000	-1.330	1003	0.184
**Slope for Spring (reference category: Winter)**					
Intercept	-0.157659	0.212157	-0.743	1003	0.458
**Slope for Summer (reference category: Winter)**					
Intercept	-0.190965	0.240435	-0.794	1003	0.427
**Slope for Autumn (reference category: Winter)**					
Intercept	0.093353	0.243804	0.383	1003	0.702

Note. Coefficient = unstandardized regression coefficient. SE = standard error of the regression coefficient; DF = degrees of freedom (df changes due to missing values or to the presence of fixed\random time slopes).

Supplementary Table S3.

Fixed effects (with robust standard errors) for the dependent variable “Fish Intake”.

Fixed Effects for Fish Intake	Coefficient	SE	*t*-ratio	DF	*p*-value
**Intercept (Pre-Event)**					
Intercept	0.881200	0.212181	4.153	260	<0.001
Age	-0.013094	0.025235	-0.519	260	0.604
Sex	-0.139162	0.416600	-0.334	260	0.739
Working Status	-0.189926	0.391848	-0.485	260	0.628
Marital Status	0.241312	0.359050	0.672	260	0.502
Education	0.380444	0.312075	1.219	260	0.224
Hypertension	0.377004	0.317366	1.188	260	0.236
Diabetes	0.332238	0.411082	0.808	260	0.420
Dyslipidaemia	-0.031932	0.308946	-0.103	260	0.918
Obesity	-0.661966	0.403469	-1.641	260	0.102
Family History of CVD	-0.511585	0.312492	-1.637	260	0.103
**Slope Time.1 (Pre- to Post-Event)**					
Intercept	0.901937	0.311413	2.896	1006	0.004
Age	0.000770	0.052542	0.015	1006	0.988
Sex	-0.216674	0.817402	-0.265	1006	0.791
Working Status	0.241351	0.793509	0.304	1006	0.761
Marital Status	-0.068228	0.670296	-0.102	1006	0.919
Education	0.651790	0.629923	1.035	1006	0.301
Hypertension	0.079465	0.663669	0.120	1006	0.905
Diabetes	-0.520213	0.826787	-0.629	1006	0.529
Dyslipidaemia	-0.206018	0.617891	-0.333	1006	0.739
Obesity	1.250195	0.891408	1.402	1006	0.161
Family History of CVD	1.305289	0.643842	2.027	1006	0.043
**Slope Time.2 (Post- to 5-years follow-up)**					
Intercept	-0.213205	0.037681	-5.658	1006	<0.001
Age	0.003561	0.005394	0.660	1006	0.509
Sex	0.016543	0.104004	0.159	1006	0.874
Working Status	-0.043635	0.089624	-0.487	1006	0.626
Marital Status	-0.095491	0.070843	-1.348	1006	0.178
Education	-0.054070	0.072753	-0.743	1006	0.458
Hypertension	0.017580	0.077207	0.228	1006	0.820
Diabetes	-0.119876	0.098454	-1.218	1006	0.224
Dyslipidaemia	-0.007508	0.072099	-0.104	1006	0.917
Obesity	-0.035468	0.107517	-0.330	1006	0.742
Family History of CVD	-0.113809	0.071542	-1.591	1006	0.112
**Slope for HADS Anxiety**					
Intercept	-0.050295	0.037286	-1.349	1006	0.178

**Slope for HADS Depression**					
Intercept	-0.014733	0.036009	-0.409	1006	0.683
**Slope for Spring (reference category: Winter)**					
Intercept	-0.250488	0.199757	-1.254	1006	0.210
**Slope for Summer (reference category: Winter)**					
Intercept	0.090167	0.204435	0.441	1006	0.659
**Slope for Autumn (reference category: Winter)**					
Intercept	0.077229	0.221635	0.348	1006	0.728

Note. Coefficient = unstandardized regression coefficient. SE = standard error of the regression coefficient; DF = degrees of freedom (df changes due to missing values or to the presence of fixed\random time slopes).

Supplementary Table S4.

Fixed effects (with robust standard errors) for the dependent variable “Red/processed Meat Intake”.

Fixed Effects for Red Meat Intake	Coefficient	SE	*t*-ratio	DF	*p*-value
**Intercept (Pre-Event)**					
Intercept	-3.707151	0.342478	-10.825	260	<0.001
Age	0.070842	0.040382	1.754	260	0.081
Sex	0.700696	0.690752	1.014	260	0.311
Working Status	-0.173341	0.613613	-0.282	260	0.778
Marital Status	-0.269076	0.630122	-0.427	260	0.670
Education	0.553276	0.520775	1.062	260	0.289
Hypertension	-0.131536	0.507795	-0.259	260	0.796
Diabetes	0.248990	0.721189	0.345	260	0.730
Dyslipidaemia	-0.661907	0.531594	-1.245	260	0.214
Obesity	-1.411057	1.105889	-1.276	260	0.203
Family History of CVD	0.454082	0.545408	0.833	260	0.406
**Slope Time.1 (Pre- to Post-Event)**					
Intercept	4.011471	0.508093	7.895	749	<0.001
Age	-0.129639	0.066672	-1.944	749	0.052
Sex	-0.911212	1.239097	-0.735	749	0.462
Working Status	0.127338	1.091556	0.117	749	0.907
Marital Status	-0.533183	1.060947	-0.503	749	0.615
Education	-0.791948	0.939026	-0.843	749	0.399
Hypertension	1.869396	0.866733	2.157	749	0.031
Diabetes	-0.767926	1.071153	-0.717	749	0.474
Dyslipidaemia	1.451101	0.901558	1.610	749	0.108
Obesity	0.891548	1.710665	0.521	749	0.602
Family History of CVD	0.164248	0.941658	0.174	749	0.862
**Slope Time.2 (Post- to 5-years follow-up)**					
Intercept	0.276420	0.043521	6.351	260	<0.001
Age	-0.001824	0.006123	-0.298	260	0.766
Sex	-0.094601	0.100962	-0.937	260	0.350
Working Status	0.069549	0.101525	0.685	260	0.494
Marital Status	0.197067	0.095766	2.058	260	0.041
Education	-0.037237	0.084255	-0.442	260	0.659
Hypertension	-0.244294	0.083858	-2.913	260	0.004
Diabetes	0.006193	0.135446	0.046	260	0.964
Dyslipidaemia	-0.037766	0.083028	-0.455	260	0.650
Obesity	0.072591	0.121317	0.598	260	0.550
Family History of CVD	-0.136685	0.084729	-1.613	260	0.108
**Slope for HADS Anxiety**					
Intercept	0.090320	0.040302	2.241	749	0.025

**Slope for HADS Depression**					
Intercept	-0.071762	0.042464	-1.690	749	0.091
**Slope for Spring (reference category: Winter)**					
Intercept	0.474112	0.196668	2.411	749	0.016
**Slope for Summer (reference category: Winter)**					
Intercept	-0.007016	0.210081	-0.033	749	0.973
**Slope for Autumn (reference category: Winter)**					
Intercept	0.434728	0.212072	2.050	749	0.041

Note. Coefficient = unstandardized regression coefficient. SE = standard error of the regression coefficient; DF = degrees of freedom (df changes due to missing values or to the presence of fixed\random time slopes).
